# Intolerance of Uncertainty and Fear of COVID-19 Moderating Role in Relationship Between Job Insecurity and Work-Related Distress in the Republic of Serbia

**DOI:** 10.3389/fpsyg.2021.647972

**Published:** 2021-06-11

**Authors:** Jelena Blanuša, Vesna Barzut, Jasmina Knežević

**Affiliations:** ^1^Department of Educational Sciences, College for Vocational Education of Preschool Teachers and Sports Trainers, Subotica, Serbia; ^2^Faculty of Sport and Tourism, Educons University, Novi Sad, Serbia

**Keywords:** job insecurity, intolerance of uncertainty, distress, fear of COVID-19, Job threats

## Abstract

The COVID-19 outbreak in Serbia was followed by strict restrictions that negatively affected the economy, particularly small size companies. The complete lockdown and the prohibition of certain services have led to an unstable employment situation. Only several studies investigated the job insecurity and its consequences during COVID-19 pandemic, and some of them highlight the fear of COVID-19 as a significant moderator of mental health. Other studies emphasize the huge effect that intolerance of uncertainty could have in explaining distress, especially during pandemic. In addition, intolerance of uncertainty was considered as a possible moderator of the relationship between the objective and subjective job threat, as well their consequences for mental health. This study aimed to examine the presence of job insecurity and work related distress in Serbia during the first wave of COVID-19. We wanted to measure the effect of the job insecurity on experienced work distress, as well the moderation potential of the intolerance of uncertainty as an individual-level and the fear of coronavirus as a situation-dependent variable. Five hundred and twenty five employed participants took part in an online study during the first wave of coronavirus infection in Serbia. To measure job insecurity, we used Perception of job insecurity scale (PJIS), while distress was assessed with Distress scale from 4DSQ. Fear of COVID-19 was measured on three items. The intolerance of uncertainty was measured by the IUS-11 scale. The results showed that 30.4% of the participants consider their employment as moderately or highly insecure, and 15.1% thought they can lose their jobs. 63.4% of participants expressed increased levels of distress. The moderation analysis revealed that the effect of job insecurity on distress can be moderated by interaction of intolerance of uncertainty and COVID-related fear. In general, distress scores were increasing with increasing job insecurity, intolerance of uncertainty and fear of COVID-19. This pattern is not observed only when fear and intolerance of uncertainty were both low, when job instability could not influence distress. This study also showed that emotional appraisal of the job threat had higher impact on distress than the perceived threat, that shed the light on the importance of considering general resilience capabilities as a protective factor in the work environment in the time of crisis.

## Introduction

The Coronavirus outbreak in 2019 made a big disturbance in the lives life of millions people around the globe. The rapid spread of the new virus, with unknown abilities and consequences, required taking numerous containment measures. These measures were based mainly on social isolation, the avoidance of gatherings and lack of physical contacts. They strongly influenced the working climate, since the majority of employees worked from home to avoid contact, or worked with changed work schedules. A number of them were also on forced leave, with the production being reduced or almost absent in certain companies. Certain non-essential businesses have been temporarily closed. At the time when this study was conducted (April 2020), the estimated mortality rate was high (Centers for Disease Control, 2020). In addition, no data were available on possible consequences and long-term negative effects. The absence from work due to illness or permanent damage to health could undoubtedly increase the risk of job loss, lower income, or search for a new job. As the result, people were not only concerned about their own health, but also about the future whiles the new economic crisis was expected to occur.

The relationship between the economic crisis and poorer mental health is well documented. In the period after the economic crisis in 2008 several European countries reported an increase in suicidal attempts (for review see Wahlbeck and McDaid, 2012). This is not surprising knowing that other studies (e.g., McKee-Ryan et al., 2005) showed that unemployment could be a risk factor for poorer mental and psychical health, lower life satisfaction, as well as lower marital satisfaction.

In this study, we aimed to examine whether the perceived job insecurity is associated with higher levels of distress during the coronavirus outbreak in Serbia. Moreover, by considering the specific situation of a pandemic, we also wanted to explore the moderation role of COVID-19 fear (a situation specific factor) and intolerance of uncertainty (as a more stable cognitive factor).

### Job Insecurity as a Predictor of Distress

Job insecurity is defined as “a personal concern about the future of the job” (van Vuuren et al., 2019, p. 230). In the past decades, this concept has been widely explored, and most of the studies showed the importance of job security for both mental health of the workers and health of the organization.

There are plenty of studies that link job insecurity to various mental health symptoms. For example, job insecurity is related to increased depression, anxiety, and somatization symptoms, as well as a decrease in well-being and life satisfaction (for review see Sverke et al., 2002; Llosa et al., 2018). A causational nature of the relationship between job insecurity on mental health has been also confirmed. A meta-analysis of 57 longitudinal studies published between 1987 and 2016 found causal effect: job insecurity was considered as a stressor which contributes to mental health problems (De Witte et al., 2016).

Job insecurity influences both general distress and work-related stress. In numerous studies, job insecurity has been identified as one of the most stressful factors in the workplace. Studies have demonstrated a direct effect of job insecurity on psychological distress, regardless of other situational, work-related and personal factors (e.g., Barnett and Brennan, 1997; Vander Elst et al., 2013; Yaşlıoǧlu et al., 2013). It emphasizes the clear connection between job insecurity and stress symptoms. Moreover, some authors view job insecurity as a direct stressor in their research (for details, see De Witte et al., 2016).

Job insecurity could be a particularly important issue during the time of COVID-19 pandemic. Study from United States (Ganson et al., 2021) have shown that job loss can worsen mental health during the coronavirus pandemic. They found that not only experienced job loss, but also expected job loss can increase anxiety, worry, depression, and to reduce interest. In fact, they found similar effects for both actual and expected employment loss among young adults. Another study (Lee, 2020) that included over 30000 participants from 27 European countries older than 50 years identified the association between job security and different aspects of mental and physical health during COVID-19 pandemic. Namely, it was shown that job security was positively associated with mental health, life satisfaction, happiness and self-assessed physical health. Moreover, job security was also associated with a reduced level of distress. Similar results were reported only 2 weeks after the measures were introduced (Pacheco et al., 2020).

Recognizing that job insecurity is a subjective estimation, even when based on an objective threat, it is important to take into consideration other factors that may influence it. Previous studies suggest at least two important factors to take into account in this context: COVID-19 related fear and the intolerance of uncertainty.

### Intolerance of Uncertainty Effect on Distress and Job Insecurity

The intolerance of uncertainty could be defined as a bias that determines how someone processes uncertain situations (Dugas et al., 2005). People with a higher intolerance of uncertainty believe that uncertainty is stressful, negative and disturbing, and that it should be avoided. Their functioning is affected in uncertain situations (Buhr and Dugas, 2002). The importance of intolerance of uncertainty as an important factor that could influence mental health has been already identified during the H1N1 pandemic (Taha et al., 2014) and confirmed in the recent COVID-19 pandemic. Several studies reported an association between the intolerance of uncertainty and stress in the general population (Bakioğlu et al., 2020; Ferreira et al., 2020).

Uncertainty can be a core concept for understanding not only the experienced distress, but also job insecurity itself. Some authors (e.g., Bordia et al., 2004) found that uncertainty could be a crucial factor during organizational change, since every change implies a lack of information and an unpredictable future. If workers cannot tolerate this lack of information and control—all together, it will result in poorer mental health and increased distress. Considering the broader concept of social climate, there is a different way of dealing with uncertainty between different societies (Debus et al., 2012). If uncertainty is avoided by enacting a wide range of laws and regulations – the individual appraisal of job loss can be less threatening. Since the high level of uncertainty in the work context was specific at the time of the pandemic, we decided to further explore its role in the relationship between job insecurity and distress.

An individual can see situation of a potential job loss as more or less threatening, depending on their capability to deal with the uncertainty of the job-loss and all its effects. Greenhalgh and Rosenblatt (1984), in their complex conceptual model that describes how job insecurity affects organizational effectiveness, have first considered the influence of personal variables on perceived job insecurity. They made the distinction between the objective threat and the subjective threat to employment. Objective threat is a consequence of the actual job-threatening situation, while subjective job threat includes a personal perception of actual threat, including the severity of the threat and a sense of powerlessness. They proposed that the relation between objective and subjective job threat can be moderated by some individual difference variables, including the need for security. They were considering intolerance of insecurity and intolerance of ambiguity as key concepts for assessing and testing the need for security role in this relationship. Both subjective and objective job threats, in interaction with other variables, could influence reactions to job insecurity.

Adkins and colleagues (Adkins et al., 2001) were the first to empirically test the relationship between job insecurity and tolerance of ambiguity (another commonly used concept that assesses personal potential to deal with uncertain situations). They found that intolerance of ambiguity was a strong predictor of job insecurity among university professors during the time of significant budget cuts. If one cannot tolerate ambiguity—job insecurity will actually be higher.

Given those studies in mind, tolerance of uncertainty/ambiguity seems to be an important concept for understanding both job insecurity and distress, and it is reasonable to question whether intolerance of uncertainty can influence their relationship. Although there are no studies that directly explored this influence, a study from Italy after the global economic crisis (Chirumbolo and Areni, 2010) examined the moderating role of similar theoretical construct—the need for closure (the wider concept that includes intolerance of ambiguity). They found a moderating role of the need for closure on the relation between job insecurity and mental health. The need for closure moderated this link in a complex way: when the need for closure was low, job insecurity affects mental health in a negative manner, while when it was high—mental health was independent of job insecurity. It gives us strong indication that the intolerance of uncertainty/ambiguity might be a significant factor in explaining the relationship between job uncertainty and mental health, but further exploring the intolerance of uncertainty’s particular role should be considered.

### The Fear of COVID-19 Moderating Potential Between Job Insecurity, Distress, and Intolerance of Uncertainty

Dealing with an unknown virus reasonably provokes fear that could affect someone’s functioning. While moderate levels of fear could motivate people to be more responsible and to follow restrictive measures, high levels of fear, especially over a longer period of time, will increase levels of distress. The relation between COVID-19 related fear and level of distress and depressive symptoms was found to be significant: increased fear of virus led to poorer wellbeing, including higher distress (Gabor et al., 2020).

It has already been confirmed that fear of the virus also affects work-related distress during the pandemic. A study conducted among Italian dentists examined the relation between job insecurity and depressive symptoms, and showed that fear of COVID-19 moderated this relation. As expected, job insecurity was associated with more depressive symptoms, but the same pattern was observed for COVID-19 fear and depressive symptoms. In addition, among participants who reported low levels of fear—the relation between job insecurity and depressive symptoms was lower (Gasparro et al., 2020).

Furthermore, the positive relation between fear of COVID-19 and intolerance of uncertainty has also been demonstrated. Fear of COVID-19 has been mediating factor between intolerance of uncertainty and mental wellbeing (Satici et al., 2020). Some authors also state that the disposition for intolerance of uncertainty affects both perceived threat and distress (Freeston et al., 2020), that led us to conclusion that fear of the virus and intolerance of uncertainty should be further treated as related concepts.

#### Aim of the Study

In this study, we aimed to explore the complex relationship between fear of coronavirus, intolerance of uncertainty, job insecurity, and distress. The first objective of this study’s was to detect the presence of job uncertainty during the first wave of COVID-19 pandemic among workers, and also the presence of distress.

Furthermore, we wanted to test: (1) moderating effect of intolerance of uncertainty, as important individual cognitive appraisal factor, on the relationship between job insecurity and distress; and (2) moderation potential of fear of coronavirus as a situation-dependent factor during the pandemic. In order to make a broader picture, we aimed to explore the complex relationship between job insecurity and distress by considering the interaction of these two moderators.

## Materials and Methods

### Participants

A total number 548 participants took part in our study. Eleven participants were excluded from the sample since they were working abroad, while 12 participants had already lost their job since the beginning of the pandemic, so they were also excluded from further analyses. A final number of 525 employed participants from The Republic of Serbia took part in a web survey. They were primarily based in the region of northern (443, 84.4%) and central (35, 6.7%) region of Serbia. 152 (29%) participants were males and 373 (71%) females. The mean age was 37.34, within the range from 18 to 65 years. 14 participants (2.7%) had completed primary school, 221 (42.1%) high school, and 290 (55.2%) had earned higher education degree. All of them were employed at the time of their participation, most of them formally (488, 93%). The majority of participants came from the private sector (307, 58.5%), followed by public sector (206, 39.2), while 12 participants (2.3%) came from non-governmental organizations or had worked as freelancers or other. This differs slightly from official national statistics according which 72.53% were employed in the private sector and 27.47% in the public sector (Statistical office of Republic Serbia, 2020b). Since the study was web-based, we found that data about exact employment industry and company size will be hard to precisely collect, so it was not controlled in our study.

A *post hoc* power analysis was carried out in order to evaluate the sample size. When we are considering three main effects and four interactions later used in the analysis, observed power of our sample were 1.00 for medium and 0.73 for small effect size detection.

### Procedure

The study was cross-sectional, web-based. A survey was distributed online during the first wave of the coronavirus outbreak in Serbia (from 5 to 16th April, 2020). It started 30 days after the first registered case of COVID-19 and approximately 20 days after declaring a state of emergency. The survey was disseminated among a variety of employment-related social media groups. In addition, students from College for Vocational Education of Preschool Teachers and Sport Trainers in Subotica distributed questionnaires among their employed acquaintances in compensation of the course credits. By clicking on agreement prior to the questionnaires, all participants gave informed consent to participate in the study.

### Instruments

*Four-dimensional symptom questionnaire* (4DSQ: Terluin et al., 2004; Serbian adaptation Kalaj et al., 2011) was developed in order to measure work related distress and symptoms in the working population. A 16-item Distress scale from 4DSQ was used for the purpose of this study, followed by a 5-point Likert scale for answering (from never to always). The scoring from the original article has been applied, changing the scale from 5-point to 3-point (“never” = 0 points, “sometimes” = 1 point, and “regularly,” “often” or “very often or constantly” = 2 points). Total score across all items was calculated, resulting in values from 0 to 32. According to the authors, scores 0–10 represented low stress, 11–20 moderately elevated stress, and 20–32 strongly elevated stress. The scale showed a high reliability on our sample (α = 0.95).

For the purpose of assessing job insecurity, we used the *Perception of Job Insecurity Scale* (PJIS, Knežević, 2015; Knežević and Krstić, 2019), previously developed for the purpose of measuring job insecurity in the Republic of Serbia. It consists of 23 items, measuring perceived job insecurity in three dimensions, two emotional and one cognitive; the intensity of threat, a sense of powerlessness, and the likelihood of job loss, respectively. Participants were asked to provide a response for each statement on a five-points Likert scale (from 1–completely disagree to 5–completely agree). Since Likelihood of job loss subscale had low reliability measures on our sample (α = 0.55), we conducted another exploratory factor analysis on our data with the permission from the author. The results revealed a somewhat different structure. All three factors were replicated, but with different loadings of particular items on each factor. The intensity of threat factor was loaded with most items (17, i.e., “Thought about staying without a job terrifies me,” “It looks like every change in my company increases risk of losing a job”) giving a reliable solution (α = 0.95). Likelihood of job loss subscale was replicated with 4 items with negative loadings, and it was named as Likelihood of job loss (α = 0.84) (i.e., “As long as my company shows interest in my knowledge and competences, I consider my employment as stable,” “As long as my company offers me new projects, I consider my employment as stable”). The sense of powerlessness was loaded with just two items, and we excluded it from further analysis because it showed low reliability (α = 0.59). Intensity of threat and Likelihood of job loss scores were calculated as the total score on corresponding items.

Additionally, prior to full PIJS scale, two independent questions were formulated in order to directly assess Worry about job loss and Worry of reducing salary (“Are you worried about losing job/reducing salary during the pandemic”) on the 5-pont scale (from “not at all” to “highly concerned”); as well one question measuring Employment stability categorically (“I consider my job as: highly unstable, moderately unstable, moderately stable, and highly stable”).

*Intolerance of uncertainty scale* (IUS; Freeston et al., 1994) represents a tendency to react negatively in uncertain situations. The scale consisted of 27 items, but Serbian adaptation and validation (IUS-11; Mihić et al., 2014) showed good metric characteristics of short solution (11-items; e.g., “Uncertainty keeps me from living a full life,” “I can’t stand being taken by surprise”). Participants evaluated whether some situation is characteristical for them (1–not at all, to 5–entirely). It consisted of two dimensions. Cognitive and emotional aspects of intolerance are measured with the Prospective anxiety dimension, while Inhibitory anxiety measures influences of the uncertainty on daily functioning. Since two subscales were highly correlated (*r* = 0.73, *p* < 0.01), only the total IU score was used in the analysis. The whole scale showed high reliability (α = 0.93).

As our study was conducted at the beginning of the pandemic, no instruments for measuring fear of COVID-19 were available at that time. Therefore, we constructed a short *Fear of COVID-19 scale* for the purpose of this study. It was based on Protection motivation theory (Rogers, 1975; Maddux and Rogers, 1983; adapted from Milne et al., 2002). The scale concluded three items assessing anxiety, worry and scare of coronavirus infection (“The thought of developing COVID-19 makes me feel anxious/worried/scared“). The mean score was calculated, with possible values from 1 to 5. The scale showed high reliability (α = 0.94).

### Statistical Analysis

Preliminary analyses were conducted in order to reveal the descriptive characteristic of all variables and sample, as well correlation between Distress, Intensity of job threat, Perceived job insecurity, Intolerance of uncertainty and Fear of COVID-19.

Kruskal Wallis test was performed in order to test differences between groups of different working pattern during pandemics on Intensity of job treat, and Wilcoxon rank sum test using Benjamini and Hochberg correction method was used to make pairwise comparisons between groups. Univariate ANOVA was used to test these between-group differences on Perceived job insecurity and Distress. Multiple linear regression analysis was carried out in order to preliminary test effects of all independent variables on Distress.

Moderation model was performed in order to test moderation effect of Intolerance of uncertainty and Fear of COVID-19 and their interaction on the relationship between Intensity of threat and Distress. Same model was used to test the relationship between Perceived job insecurity and Distress and moderating effects of IU and Fear of Covid. To test these moderation effects, we used the model 3 of the PROCESS macro made by Hayes (2018). All the analyses were carried out in the R environment for statistical computing.

## Results

On the average, participants reported a moderately-high levels of Distress (15.57 out of 32). Based on the original scoring, 174 participants (33.1%) expressed strongly elevated levels of distress, 159 (30.3%) moderately elevated and 192 participants (36.6%) were in no distress category.

Intolerance of uncertainty was 8.79 points higher than in the previous study on a Serbian population (*M* = 19.11, *SD* = 6.34, Sokić et al., 2012). The mean score of Fear of COVID-19 (2.85 out of 5) can be considered as a medium-sized. The mean score of the Intensity of job threat (35.9 out of 85) and Likelihood of job loss (9.49 out of 20) can be considered as a lower-medium. All descriptive results are shown in Table 1.

**TABLE 1:** Descriptive statistics of all variables used in research.

Scale	N	Min	Max	Mean	SD	Skewness	Kutrosis
Age	525	18	65	37.34	11.23	0.16	–0.95
DSQ–distress	525	0	32	15.57	9.80	0.15	–1.18
Intensity of job threat (PJIS)	525	17	85	35.9	16.27	0.74	–0.29
Likelihood of job loss (PJIS)	525	4	20	9.49	4.56	0.61	–0.46
IUS-11–Intolerance of uncertainty	525	11	55	27.94	10.60	0.48	–0.34
Fear of COVID-19	525	1	5	2.85	1.28	0.07	–1.08

To examine the presence of job insecurity in more detail, we analyzed three items preceding PIJS scale (Employment stability, Worry about job loss, and Worry about reducing salary). Frequency and descriptive analysis showed concerning results. During the time of a pandemic, 39 (7.4%) participants consider their workplace as highly, and 121 (23%) as moderately unstable. Seventy nine participants (15.1%) were concerned about job loss (*M* = 2.10, *SD* = 1.29). More than a job loss, participants were much more concerned about the wage reductions during the time of a pandemic (152, 28.9% was highly or moderately concerned; *M* = 2.54, *SD* = 1.39).

Considering the style of employment during the pandemic, 81 participants (15.4%) worked with regular, and 140 (26.7%) with changed working hours, while 195 participants (37.1%) worked entirely from home. On hundred and nine participants (20.8%) did not work at all at the time of study, because they were on the forced leave. In order to compare Intensity of job threat, Job stability and Distress among groups with different styles of employment during pandemic, we performed separate between-group analyses. Intensity of job threat was tested using Kruskal–Wallis test since we found a violation of the equality of error variances assumption. The groups differed in the perceived Intensity of job threat [*H*_(3)_ = 14.10, *p* < 0.01]. Participants on forced leave had highest mean rank on Intensity of threat, and Wilcoxon rank sum test using Benjamini and Hochberg correction method revealed that they differed significantly from all other groups. In addition, difference between those who are working from home and those who are working with changed working hours was found (mean ranks are presented in Table 2). Likelihood of job loss and Distress were tested using separate one-way ANOVAs, which revealed no differences between groups neither for Likelihood of job loss [*F*_(3, 521)_ = 2.54, *p* = 0.06] nor Distress [*F*_(3, 521)_ = 2.26, *p* = 0.08].

**TABLE 2:** Differences between groups of different working conditions during pandemic on intensity of threat, Likelihood of job loss and Distress scales.

		Intensity of job threat		Likelihood of job loss	Distress
				
	N (%)	Mean (SD)	Mean rank	Mean (SD)	Mean (SD)
Regular hours	81 (15.4)	34.79 (15.5)	254.97	8.60 (4.44)	15.53 (10.03)
Changed hours	140 (26.7)	33.13 (16.38)	231.30	9.31 (4.73)	14.26 (9.59)
Work from home	195 (37.1)	34.49 (14.33)	266.65	9.47 (4.37)	15.45 (9.35)
On forced leave	109 (20.8)	41.02 (18.84)	303.19	10.39 (4.61)	17.50 (10.50)
Total		35.90 (16.27)		9.49 (4.56)	15.57 (9.8)

A correlation analysis was performed to explore the relationship between all variables (all results are presented in Table 3). The strongest correlation with Distress was obtained for Intolerance of uncertainty (*r* = 0.58, *p* < 0.01), and it was medium-strong in size. Intensity of job threat and Fear of COVID-19 were both moderately correlated with Distress, while Likelihood of job loss was slightly but significantly correlated with it.

**TABLE 3:** Intercorrelations of all variables used in research.

	1	2	3	4
1. Distress				
2. Intensity of threat	0.39**			
3. Likelihood of job loss	0.20**	0.20**		
4. Intolerance of uncertainty	0.58**	0.38**	0.18**	
5. Fear of COVID-19	0.37**	0.20**	0.05	0.43**

**p < 0.01.

Intensity of threat was positively correlated with Intolerance of uncertainty, while the correlation with Fear of COVID-19 was low and significant. On the other hand, Likelihood of job loss had low, positive and significant correlations with both Distress and Intolerance of uncertainty, while its correlation with fear of COVID-19 was almost absent. It can also confirm the importance of differentiating cognitive and emotional aspects of job insecurity, especially because of their low negative intercorrelation.

Two separate multiple regression analyses were performed in order to test effects of two Perception of job insecurity scales together with other predictors (IU and Fear of COVID-19) on Distress. Both models revealed significant results with all significant predictors (see Tables 4A,B).

**TABLE 4:** Two regression models with different PJIS predictors: **(A)** Intensity of threat and **(B)** Job stability.

Regression model	Predictors	*B*	SE	*b*	*t*	*P*
**(A)** *R*^2^ = 0.39, *F*_(__3, 521)_ = 109.47, *p* < 0.001	Intensity of threat	0.11	0.02	0.19	5.01	0.000
	Intolerance of uncertainty	0.42	0.04	0.45	11.30	0.000
	Fear of COVID-19	1.02	0.29	0.14	3.94	0.001
**(B)** *R*^2^ = 0.37, *F*_(__3, 521)_ = 101.08, *p* < 0.001	Job stability	0.23	0.08	0.11	2.98	0.003
	Intolerance of uncertainty	0.46	0.04	0.50	12.79	0.000
	Fear of COVID-19	1.12	0.30	0.15	3.78	0.000

**FIGURE 1:**
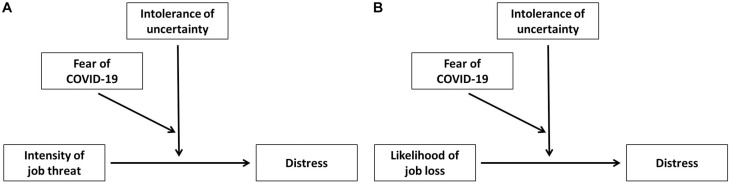
Path diagram describing intolerance of uncertainty and fear of COVID-19 moderation in relationship between **(A)** intensity of job threat and distress and **(B)** likelihood of job loss and distress.

Further, we aimed to test moderation effect of Intolerance of uncertainty and Fear of COVID-19 on the relationship of Intensity of threat and Distress, on the one hand, and on relationship of Job stability and Distress, on the other hand. We performed a moderation analysis with the interaction of two moderators. Path diagrams are presented on Figures 1A,B.

### Intensity of Threat

The results revealed a significant main effect of Fear of COVID-19, making in most important independent contributor, while Intolerance of uncertainty and Intensity of threat were not significant. The exact nature of the relationship between these variables can be seen through their interactions (see Table 5).

**TABLE 5:** Results of moderated moderation analysis with Intensity of threat as a predictor.

	B	se	t	p	LLCI	ULCI
Constant	9.03	4.77	1.89	0.059	–0.34	18.40
**Main effects**						
Intensity of threat (IoT)	–0.21	0.12	–1.71	0.089	–0.45	0.03
Intolerance of uncertainty (IU)	0.06	0.17	0.37	0.710	–0.27	0.40
Fear of COVID-19 (FearCov)	–3.29	1.65	–2.00	0.046	–6.53	–0.06
**Two-way interactions**						
Iot × IU	0.01	0.00	2.54	0.011	0.00	0.02
IoT × FearCov	0.13	0.04	2.97	0.003	0.04	0.21
IU × FearCov	0.13	0.05	2.50	0.013	0.03	0.24
**Three-way interaction**						
IoT × IU × FearCov	0.00	0.00	–3.06	0.002	–0.01	0.00
**Control**						
Age	–0.07	0.03	–2.37	0.018	–0.13	–0.01
Gender	0.76	0.75	1.02	0.308	–0.71	2.24
**Model**						
*R*^2^ = 0.40***						
Δ *R*^2^ = 0.01**						

**p < 0.01; ***p < 0.001.

Significant interaction *Intensity of threat x Intolerance of uncertainty* showed that with increasing both job threat and IU distress scores became higher. The same is true for *Intensity of threat x Fear of COVID-19*.

**FIGURE 2:**
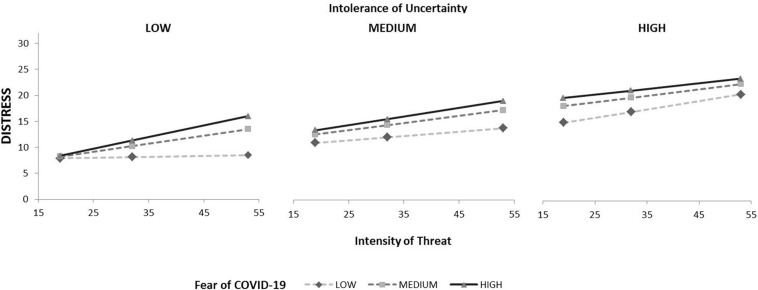
Interaction of intolerance of uncertainty, fear of COVID-19 and intensity of treat affect distress score.

Significant three-way interaction *Intensity of threat x Intolerance of uncertainty x Fear of COVID-19* showed the compound effect of the predictor and both moderators. Distress scores were increasing with increasing Intensity of threat, Intolerance of uncertainty and Fear of COVID-19. Results are presented graphically in Figure 2.

The test of conditional effects showed patterns of differences on each level of moderator (Table 6). In the group of low Intolerance of uncertainty and low Fear, Distress remained low regardless of Intensity of threat. In all other cases, distress scores have become higher as Intensity of threat, Intolerance of uncertainty and Fear were increasing.

**TABLE 6:** Conditional effects of Intensity of threat at values of two moderators.

Intolerance of uncertainty	Fear of COVID-19	*B*	*SE*	*t*	*p*	LLCI	ULCI
16.16	1	0.02	0.05	0.37	0.709	−0.08	0.12
16.16	3	0.16	0.04	4.09	0.000	0.08	0.23
16.16	4	0.22	0.06	4.06	0.000	0.12	0.33
27	1	0.09	0.04	2.14	0.033	0.01	0.16
27	3	0.14	0.03	5.63	0.000	0.09	0.19
27	4	0.17	0.04	4.71	0.000	0.10	0.24
39	1	0.16	0.05	3.01	0.003	0.06	0.26
39	3	0.13	0.03	4.66	0.000	0.07	0.18
39	4	0.11	0.03	3.58	0.000	0.05	0.17

### Likelihood of Job Loss

Moderated moderation analysis was also performed with Likelihood of job loss as predictor, Intolerance of uncertainty and Fear of COVID-19 as moderators, and Distress as criterion variable. Results revealed somewhat different structure (presented in Table 7).

**TABLE 7:** Results of moderated moderation analysis with Likelihood of job loss as a predictor.

	*B*	*SE*	*t*	*p*	LLCI	ULCI
Constant	5.03	4.64	1.08	0.279	–4.09	14.15
**Main effects**						
Likelihood of job loss (LJL)	–0.54	0.41	–1.32	0.188	–1.35	0.27
Intolerance of uncertainty (IU)	0.20	0.17	1.16	0.247	–0.14	0.54
Fear of COVID-19 (FearCov)	–1.08	1.55	–0.69	0.488	–4.13	1.98
**Two-way interactions**						
LJL × IU	0.03	0.02	1.87	0.062	0.00	0.06
LJL × FearCov	0.26	0.15	1.78	0.076	–0.03	0.55
IU × FearCov	0.08	0.05	1.53	0.127	–0.02	0.18
**Three-way interaction**						
LJL × IU × FearCov	–0.01	0.00	–1.98	0.049	–0.02	0.00
**Control**						
Age	–0.06	0.03	–1.97	0.050	–0.12	0.00
Gender	0.76	0.77	0.99	0.325	–0.75	2.26
**Model**						
*R*^2^ = 0.38***						
Δ *R*^2^ = 0.005*						

*p < 0.05; ***p < 0.001.

**FIGURE 3:**
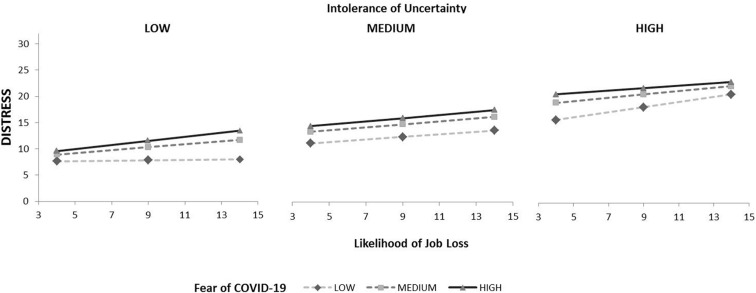
Interaction of intolerance of uncertainty, fear of COVID-19 and likelihood of job loss affect distress score.

When we are focusing on Likelihood of job loss as a predictor, none of the main effects or two way interactions were significant, indicating that Likelihood of job loss, Intolerance of uncertainty and Fear of COVID cannot explain Distress by themselves. However, the marginally significant three-way interaction of moderators and predictor showed that both moderators and predictor working together can influence distress score. Results are presented graphically in Figure 3.

Considering conditional effects (see Table 8), we found that the predictor in interaction with both moderators lead to increased Distress, except in two cases: when Fear was low and IU was low, as well when Fear was low and IU was medium in size. In those cases, Distress score is independent of Likelihood of job loss.

**TABLE 8:** Conditional effects of Perceived job insecurity at specific values of two moderators.

Intolerance of uncertainty	Fear of COVID-19	*B*	*SE*	*t*	*p*	LLCI	ULCI
16.16	1	0.038	0.151	0.252	0.801	–0.259	0.335
16.16	3	0.275	0.133	2.062	0.04	0.013	0.536
16.16	4	0.393	0.196	1.999	0.046	0.007	0.779
27	1	0.251	0.134	1.875	0.061	–0.012	0.515
27	3	0.295	0.088	3.371	0.001	0.123	0.468
27	4	0.317	0.126	2.513	0.012	0.069	0.566
39	1	0.487	0.222	2.198	0.028	0.052	0.923
39	3	0.319	0.112	2.855	0.004	0.099	0.538
39	4	0.234	0.114	2.055	0.04	0.01	0.458

## Discussion

Studies that examined the effect of COVID-19 pandemic on working population were mostly focused on healthcare workers as one of the most vulnerable groups (Gasparro et al., 2020; Giorgi et al., 2020; Mattila et al., 2021). One study in general working population in Finland has shown that COVID-19 anxiety was associated with perceived loneliness, neuroticism, distress (Savolainen et al., 2021). There is an evident lack of information how workers in different professions and those with more unstable job and income situation are dealing with all changes and uncertainty during a COVID-19 pandemic.

The restrictive measures taken in Serbia did not differ substantially from those taken in the rest of Europe. However, the Serbian job market situation is specific – unstable. During the first quarter of 2020 (Statistical office of Republic Serbia, 2020a) less than a half of the working population (48.7%) was formally employed, 16.2%. of the population with informal employment, while the unemployment rate was 9.7%. Finally, there is no information about the status of one quarter of residents (25.4%), perhaps because they are inactive, i.e., not looking for a job. Therefore, even if was unknown whether this situation will be temporary or long-lasting, we assumed that pandemic will have an immediate negative impact on the economy and increase job loss and job insecurity (while this impact might be delayed in countries with more stable economy).

Our study found that 15.1% of employees in our sample were concerned about losing their employment during the first wave of the COVID-19 pandemic. Moreover, at least 12 participants in our initial sample (2.23%) had already lost their job by the end of the study (1 month after the introduction of restrictions). In other words, almost one fifth of people (17.33%) were facing real or expected job loss, and almost one third of participants considered their workplace as unstable. Such a result clearly indicates that workers experienced an increased sense of job threat during the first wave of the coronavirus pandemic. Additionally, our study showed that 1/3 of workers was experiencing high levels of distress, while intolerance of uncertainty was also increased.

Comparable to data from the United States which showed that about 25% workers cannot work from home due to the nature of the job (Baker, 2020), 20.8% of participants in our sample has already been on forced or paid leave during the time of study. Our results showed that this group is especially vulnerable to develop a sense of job threat (but not Likelihood of job loss and distress). Possible mental health consequences on this population should be further explored.

This study identified several factors related to distress in working population. In first place, there are variables related to employment status: perceived job insecurity and intensity of job threat. The findings are in line with previous studies, prior to and during the pandemic, which showed that job insecurity was associated with distress (e.g., De Witte, 1999; Popov et al., 2010; Lee, 2020). In addition, we found a moderation effect of intolerance of uncertainty as an individual characteristic, and fear of COVID-19 which was a situation-dependent variable.

Similarly to Gasparro et al. (2020), we found that effect of job insecurity on distress would not be significant if fear of coronavirus is low. Moreover, including additional moderator—intolerance of uncertainty—enabled us to detect the more precise pattern of their relationship. We found true that job insecurity would not influence distress in the case when both fear and intolerance of uncertainty were low. In all other cases, job insecurity, intolerance of uncertainty and fear contribute all together to increased distress levels. This revealed that job insecurity does not contribute to distress if someone does not find a virus threatening and if he tolerates uncertainty well, but otherwise does.

Our study design is partially comparable also with Chirumbolo and Areni’s (2010) who found that the need for closure moderately links job insecurity and distress. However, we did not replicate their findings. While they found that job insecurity affects mental health only when the need for closure is low, we found just the opposite: that job insecurity affects distress generally, except when intolerance of uncertainty was low and fear was low. Opposite results could be a consequence of using different constructs to measure dealing with uncertainty; namely, they used the need for closure that is a wider concept which includes intolerance of uncertainty/ambiguity, but also the preference for order and structure, the need for predictability, closed-mindedness and decisiveness, who also might influence mental health. Obtaining different pattern of results could also be due to different socio-economic circumstances: their study was conducted during the economic crisis, while ours was conducted during a health crisis. In a health crisis, losing a job in not only concern—there is a concern for health, as well. This concern revealed to be important, since we found it could moderate the relationship between job insecurity and distress.

It is important to consider two aspects of measuring job insecurity, cognitive (Likelihood of job loss) and emotional (intensity of job threat) in our study. Although they were slightly correlated, and behaving differently in our study, the final results are similar for both of them, indicating the three-way interaction of job instability, intolerance of uncertainty and fear of coronavirus on distress. However, when the cognitive aspect was assessed, job insecurity effect on distress is smaller and marginally significant; indicating that the emotional dimension of job instability could predict distress much better. It is a very indicative result, revealing that someone’s personal appraisal of job threatening event will explain distress better than perceived presence of the threat itself. In other words, our results are suggesting that even in a more stable society with better workers’ rights and protection, individuals with higher intolerance of uncertainty might experience higher job insecurity and all related negative psychological outcomes. Therefore, the role of intolerance of uncertainty in relation between job insecurity and experienced distress should be further explored in other countries with different socio-demographic circumstances.

### Strengths and Limitations of Present Study

To our knowledge, this study was the first to test both cognitive appraisal variables (intolerance of uncertainty) and situation-dependent variable (fear of COVID-19) as moderators in the relationship between job insecurity and distress. Taking both aspects together seems especially important at the time of a pandemic, when general fear of infection can override the influence of work-related variables on distress. On the other hand, the fear of the infection seems to be reasonable concern also in work context—if someone is ill, salary can be reduced, but threat of loosing work position could be possible consequence of prolonged leave, also.

Moreover, our study is first to test intolerance of uncertainty moderation role between job insecurity and distress, as Greenhalgh and Rosenblatt (1984) proposed in their model. It made place considering this important individual appraisal variable when we are estimating effects of job threat on mental health, but as well on other work-functioning aspects.

However, this study has several limitations. First of all, it is the cross-sectional study; therefore long-term effects cannot be measured. In addition, our study was correlational in nature, and that precise causal relationship between variables should be further explored. Furthermore, our sample may not represent well whole Serbian workers population, since the participants were recruited via social networks and a snowball method. Not only regionally biased, but also those who are more concerned about their job might be more likely to take part in the study. Also, since study was conducted online, some of the workers who are not using social networks were omitted (especially older). Finally, we did not measure specific organizational variables that might also take part in job insecurity (company size, industry etc.).

## Conclusion

Generally speaking, our study revealed that job insecurity affects distress, but this relationship was moderated with intolerance of uncertainty and fear of infection: individuals with a lower tolerance for uncertainty and higher fear would experience higher distress levels with increasing job insecurity.

The main contribution of this study is the fact that we showed that job insecurity does not necessarily reflect only the stability of a particular society during health crisis, but, rather, could reflect someone‘s ability to tolerate uncertainty in general. Therefore, presented result might not be limited only to Serbian workers and should be further explored in countries with different socio-economic circumstances.

The role of fear and intolerance of uncertainty regarding perceived job insecurity could be used as a standpoint for building resilience in a work context, and it leaves a room for practitioners to develop programs that can increase tolerance of uncertainty but also provide a better guideline for managing stress. In times of crisis and change, uncertainty and fear are inevitable, so we could work on the development of the individual capacities for dealing with it. Other coping mechanisms that can mediate between job insecurity and distress should be assessed in some other research in order to give a full picture about possible protective mechanisms.

## Data Availability Statement

The raw data supporting the conclusions of this article will be made available by the authors, without undue reservation.

## Ethics Statement

Ethical review and approval was not required for the study on human participants in accordance with the local legislation and institutional requirements. The patients/participants provided their written informed consent to participate in this study.

## Author Contributions

All authors listed have made a substantial, direct and intellectual contribution to the work, and approved it for publication.

## Conflict of Interest

The authors declare that the research was conducted in the absence of any commercial or financial relationships that could be construed as a potential conflict of interest.
